# Comparing vorticity and curvature Rossby numbers

**DOI:** 10.1016/j.fmre.2025.03.004

**Published:** 2025-03-13

**Authors:** Chuanyin Wang, Rui Xin Huang, Dake Chen, Qinghua Yang

**Affiliations:** a School of Atmospheric Sciences, Sun Yat-sen University, and Southern Marine Science and Engineering Guangdong Laboratory (Zhuhai), Zhuhai 519082, China; b Department of Physical Oceanography, Woods Hole Oceanographic Institution, Woods Hole, MA 02543, USA; c State Key Laboratory of Satellite Ocean Environment Dynamics, Second Institute of Oceanography, Ministry of Natural Resources, Hangzhou 310012, China

**Keywords:** Nonlinear, Rossby number, Natural coordinate, Curvature, Multiscale, Submesoscale

## Abstract

In ocean dynamics, there is often a need to measure the point-by-point significance of the nonlinear term compared with the Coriolis term in the momentum equations. The bulk Rossby number (i.e., $\frac{U}{fL}$) does not meet this need, which necessitates the proposal for the pointwise Rossby number. Conventionally, two different formulations are used to represent the pointwise Rossby number approximately. One is the vorticity Rossby number defined as the ratio of the relative vorticity to the planetary vorticity (i.e., $\frac{\zeta }{f}$), and the other is the curvature Rossby number formulated as the ratio of the curvature vorticity to the planetary vorticity (i.e., $\frac{\zeta_{curv}}{f}$). It remains unknown which approximate representation is more accurate. Here we compare their accuracies on the basis of theoretical and data analysis. The vorticity Rossby number is found to overestimate the pointwise nonlinearity of oceanic flows due to its neglect of the spatial variation of the kinetic energy. The curvature Rossby number is shown to intrinsically consider the kinetic energy term; thus, it is more accurate and useful for diagnosing and understanding the nonlinearity of ocean circulation.

## Graphical Abstract

**Figure:**
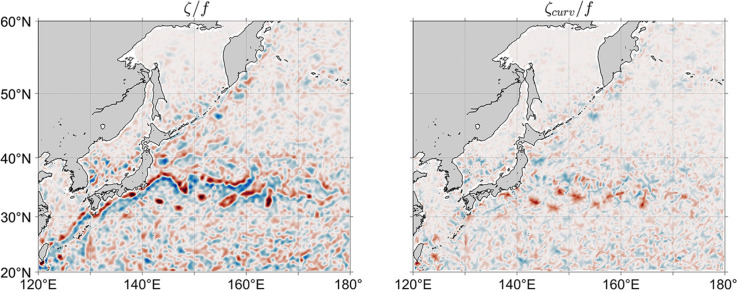

## 1 Introduction

The momentum equations describing the oceanic flow involve many terms, such as the temporal derivative, nonlinear advection, Coriolis acceleration, pressure gradient force and viscosity. Non-dimensional numbers are often introduced to evaluate the importance of one term in comparison with others. For example, in the textbooks of geophysical fluid dynamics [[1], [2], [3]], the relative importance of the advection and Coriolis terms is estimated by the bulk Rossby number *Ro_bulk_* $=\;\frac{U}{fL}$, where *U, L* and *f* are the characteristic velocity scale, length scale and Coriolis parameter, respectively. When *Ro_bulk_* is much smaller than one for a dynamical process (e.g., barotropic tide), the advection term can be neglected and the linear dynamics dominates; when *Ro_bulk_* is on the order of one for a certain type of oceanic motion (e.g., submesoscale process), advection and Coriolis terms are comparable so that both terms should be retained; when an oceanic process (e.g., three-dimensional turbulence) is characterized by *Ro_bulk_* much larger than one, the Coriolis term is negligible and it is a highly-nonlinear regime. Unfortunately, in practice, it is nontrivial to specify characteristic scales *U* and *L* in calculating *Ro_bulk_* because the oceanic motion is a nonlinear entanglement of multiple dynamical processes, ranging from the basin scale through the mesoscale to the dissipation scale. Moreover, by definition, *Ro_bulk_* is a rough estimate for an oceanic regime rather than an accurate representation of the nonlinearity. Thus, it is very useful for the scale and asymptotic analysis, but it is less useful when there exists the necessity to accurately represent the point-by-point nonlinearity. This necessity is becoming even more pressing with the rapid development in the study aimed at submesoscale processes embedded in the highly variable, multiscale ocean circulation. A pointwise Rossby number is desirable for the local and accurate comparison of the advection and Coriolis terms.

Usually invoked, without any justification, as an approximation of the pointwise Rossby number is the gradient Rossby number $\frac{\zeta }{f}$, where ζ is the vertical component of the relative vorticity (hereafter referred to as the relative vorticity) and *f* is also known as the planetary vorticity [4,5]. In this study, the gradient Rossby number is called the vorticity Rossby number, namely *Ro_vort_* $=\;\frac{\zeta }{f}$, for a comparison with the other formulation of the pointwise Rossby number, which will be introduced in the next paragraph. We think that *Ro_vort_* is a gradient non-dimensional number that finds its greatest use in the stability analysis (e.g., inertial stability); thus, a large *Ro_vort_* does not necessarily imply the significance of the advection term. An insightful example is the turbulent thermal wind, which is a linear horizontal momentum balance among the Coriolis force, baroclinic pressure gradient and vertical momentum mixing [6,7]. For a filament in the turbulent thermal wind balance, *Ro_vort_* can reach as large as 5.3 [6,7]. This example demonstrates that *Ro_vort_* is not a dynamically accurate approximation of the pointwise Rossby number. One aim of this study is to comprehensively elucidate why this could happen.

Another approximation of the pointwise Rossby number is proposed as $\frac{\zeta_{curv}}{f}$, where $\zeta_{curv}$ is the curvature vorticity [[8], [9], [10]]. In this study, we call it the curvature Rossby number, namely *Ro_curv_* $=\;\frac{\zeta_{curv}}{f}$. Although it has been introduced for a long time, *Ro_curv_* receives less attention than does *Ro_vort,_* and it remains unclear how accurate *Ro_curv_* is, in comparison with *Ro_vort_*, in measuring the pointwise nonlinearity strength. Our second aim is to show that *Ro_curv_* is a better candidate for the pointwise Rossby number.

In Section 2, the vorticity Rossby number is derived from the momentum equations in the Cartesian coordinate system, and its shortcomings are illustrated with the help of idealized flow models. In Section 3, the curvature Rossby number is formulated based on the momentum equations in the natural coordinate system, and its advantages are demonstrated in comparison to the vorticity Rossby number. Section 4 further compares these two Rossby numbers by applying them to the analysis of satellite altimetric observations and high-resolution numerical simulation. Section 5 concludes the paper with a summary and discussion.

## 2 Vorticity Rossby number

### 2.1 Mathematical derivation

Consider the rotating shallow-water momentum equations in the Cartesian coordinate system:

(1) $$\left\{ \begin{array}{c} \frac{\partial u}{\partial t}+u\frac{\partial u}{\partial x}+v\frac{\partial u}{\partial y}-fv=-g\frac{\partial \eta }{\partial x}\\ \frac{\partial v}{\partial t}+u\frac{\partial v}{\partial x}+v\frac{\partial v}{\partial y}+fu=-g\frac{\partial \eta }{\partial y} \end{array}\right.$$

where u is the *x*-direction velocity, v the *y*-direction velocity, g the acceleration due to gravity and η the sea surface height. Let u=(u,v), $\nabla_{h}=\left(\frac{\partial }{\partial x},\frac{\partial }{\partial y}\right)$ and ζ=(0,0,ζ). Using the vector identity $u\cdot \nabla_{h}u$ $=\nabla_{h}\left(u\cdot u\right)/2+\zeta \times u$, the nonlinear advection terms of Eq. 1 can be rewritten into the well-known Gromeka-Lamb form [1,11]:

(2) $$\left\{ \begin{array}{c} u\frac{\partial u}{\partial x}+v\frac{\partial u}{\partial y}=-\zeta v+\frac{1}{2}\frac{\partial \left(u^{2}+v^{2}\right)}{\partial x}\\ u\frac{\partial v}{\partial x}+v\frac{\partial v}{\partial y}=\zeta u+\frac{1}{2}\frac{\partial \left(u^{2}+v^{2}\right)}{\partial y} \end{array}\right.$$

where $\zeta =\frac{\partial v}{\partial x}-\frac{\partial u}{\partial y}$ is the relative vorticity. Obviously, both the relative vorticity and the spatial inhomogeneity of the kinetic energy contribute to the nonlinearity of the momentum equations. Correspondingly, Eq. 1 becomes:

(3) $$\left\{ \begin{array}{c} \frac{\partial u}{\partial t}-\left[\zeta v-\frac{1}{2}\frac{\partial \left(u^{2}+v^{2}\right)}{\partial x}\right]-fv=-g\frac{\partial \eta }{\partial x}\\ \frac{\partial v}{\partial t}+\left[\zeta u+\frac{1}{2}\frac{\partial \left(u^{2}+v^{2}\right)}{\partial y}\right]+fu=-g\frac{\partial \eta }{\partial y} \end{array}\right.$$

By definition, the pointwise Rossby number is the ratio of the nonlinear advection term to the Coriolis acceleration term, yielding:

(4) $$\left\{ \begin{array}{c} Ro_{x}=-\frac{1}{fv}\left(u\frac{\partial u}{\partial x}+v\frac{\partial u}{\partial y}\right)=\frac{\zeta }{f}+\frac{1}{-fv}\frac{1}{2}\frac{\partial \left(u^{2}+v^{2}\right)}{\partial x}\\ Ro_{y}=\frac{1}{fu}\left(u\frac{\partial v}{\partial x}+v\frac{\partial v}{\partial y}\right)=\frac{\zeta }{f}+\frac{1}{fu}\frac{1}{2}\frac{\partial \left(u^{2}+v^{2}\right)}{\partial y} \end{array}\right.$$

where *Ro_x_* and *Ro_y_* denote the pointwise Rossby number in *x*- and *y*-momentum equations, respectively. It is evident that the vorticity Rossby number *Ro_vort_* merely includes the effect of the relative vorticity and neglects the contribution from the spatial variation of the kinetic energy. As a result, the pointwise Rossby number is expected to be significantly misestimated by *Ro_vort_* wherever the kinetic energy varies significantly. In the following analysis, four idealized flow models will be used to demonstrate that the kinetic energy term indeed substantially counteracts the effect of the relative vorticity and thus plays an important role in regulating the nonlinearity of the momentum equations (i.e., Eq. 1 or 3).

### 2.2 Idealized flow models

**Fig. 1:**
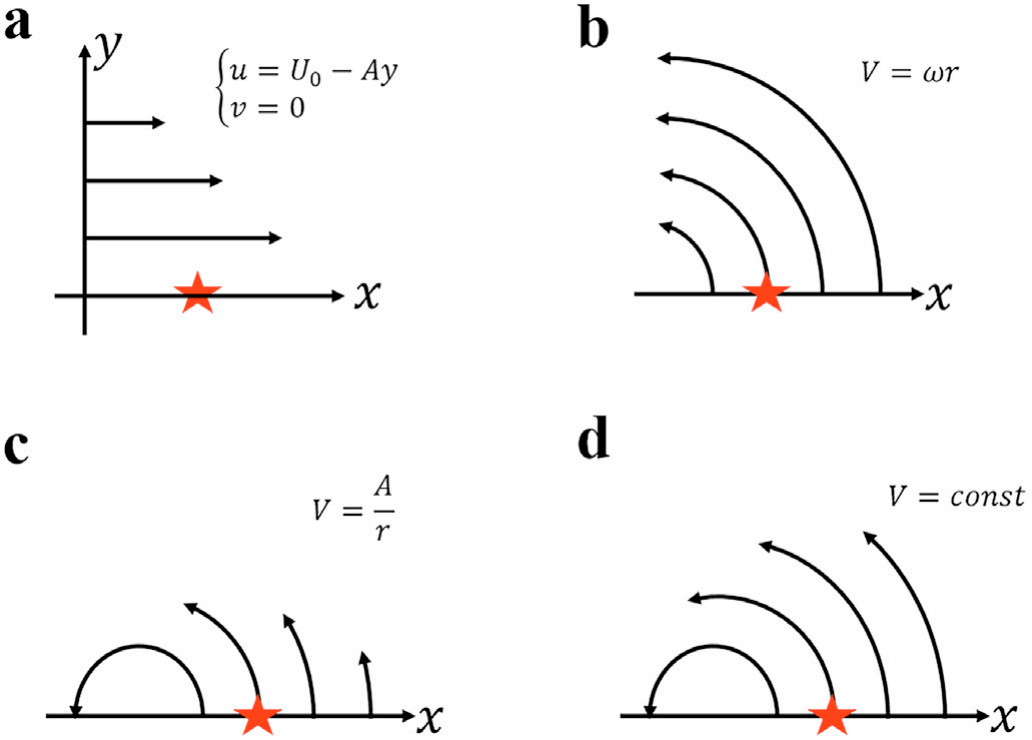
**Idealized flow models.** Panels (a-d) show the uniform shear flow, uniform circular motion, point vortex, and circular motion with uniform kinetic energy, respectively. The solid lines represent streamlines, with arrows indicating flow directions. The red pentagrams denote the locations under consideration. *U_0_* in (a) is a constant flow along the positive *x* direction, *A* in (a, c) is a positive constant, *V* in (b-d) is the flow speed, ω in (b) is the angular speed, and *r* in (b, c) is the radial distance.

First, the shear flow (e.g., the Kuroshio, the Antarctic Circumpolar Current) is one of the most common motions in the ocean and deserves particular attention. As shown in Fig. 1a, the velocity profile of the uniform shear flow model is $\left\{ \begin{array}{c} u=U_{0}-Ay\\ v=0 \end{array}\right.$, where *U_0_* and *A* are constant. From Eq. 3, we find that the *x*-momentum equation is reduced to a trivial identity 0 = 0 at the location denoted by the red pentagram, while the *y*-momentum equation becomes:

(5a) $$\left[\left(-\frac{\partial u}{\partial y}\right)u+\frac{1}{2}\frac{\partial u^{2}}{\partial y}\right]+fu=-g\frac{\partial \eta }{\partial y}$$

(5b) $$\Rightarrow fu=-g\frac{\partial \eta }{\partial y}$$

Note that the relative vorticity term (i.e., the first term on the left-hand side of Eq. 5a) exactly cancels the kinetic energy term (i.e., the second term on the left-hand side of Eq. 5a). As a result, the nonlinear advection term vanishes and the simple geostrophic balance is obtained. Correspondingly, *Ro_x_* in Eq. 4 is ill-defined because each term of the *x*-momentum equation is identically zero, and the *y*-direction pointwise Rossby number becomes:

(6) $$Ro_{y}=0$$

Eq. 6 indicates a simple fact that the geostrophic balance has a vanishing pointwise Rossby number. In striking contrast, the vorticity Rossby number *Ro_vort_* $=\;\frac{\zeta }{f}=\;-\frac{1}{f}\frac{\partial u}{\partial y}\;=\;\frac{A}{f}$ is non-zero and is thus unable to correctly represent the pointwise nonlinearity of the momentum equations for uniform shear flows owing to the neglect of the kinetic energy term whose effect is to cancel the contribution of the relative vorticity term.

Second, the uniform circular motion (Fig. 1b) is considered because the real ocean is ubiquitously populated by vortex motions, and the mesoscale vortex circulates approximately as a rigid body [12]. The analytic velocity expression for this idealized flow model is V=ωr, where $V=\sqrt{u^{2}+v^{2}}$ is the flow speed, ω the angular speed, and *r* the radial distance. At the specific point denoted by the red pentagram, the *y*-momentum equation is identically satisfied, and this could be readily justified by introducing the polar coordinate system and substituting the velocity profile; then the *x*-momentum equation is:

(7a) $$-\left[\zeta v-\frac{1}{2}\frac{\partial (u^{2}+v^{2})}{\partial x}\right]-fv=-g\frac{\partial \eta }{\partial x}$$

(7b) $$\Rightarrow -\left[\frac{2v}{r}v-\frac{v^{2}}{r}\right]-fv=-g\frac{\partial \eta }{\partial x}\Rightarrow -\frac{v}{r}v-fv=-g\frac{\partial \eta }{\partial x}$$

Eq. 7b shows that half of the relative vorticity term is cancelled by the kinetic energy term, and the other half contributes to the centripetal acceleration, which results in the gradient wind balance. Correspondingly, the *x*-direction pointwise Rossby number in Eq. 4 becomes:

(8) $$Ro_{x}=\frac{v}{fr}$$

By contrast, the vorticity Rossby number is *Ro_vort_* $=\;\frac{\zeta }{f}\;=\;\frac{2\omega }{f}\;=\;\frac{2v}{fr}$ and gives an overestimate by a factor of 2 due to neglecting the effect of the kinetic energy term.

Third, the irrotational motion is worth being considered for the theoretical clarity. An example is the point vortex (Fig. 1c), whose relative vorticity is zero everywhere except for the singular point at the origin. For the point vortex model, the analytic velocity expression is $V\;=\;\frac{A}{r}$. Similar to the uniform circular motion, only the *x*-momentum equation is present at the specific point denoted by the red pentagram

(9a) $$-\left[-\frac{1}{2}\frac{\partial (u^{2}+v^{2})}{\partial x}\right]-fv=-g\frac{\partial \eta }{\partial x}$$

(9b) $$\Rightarrow -\frac{v^{2}}{r}-fv=-g\frac{\partial \eta }{\partial x}$$

Eq. 9b shows the gradient wind balance as in the last case, but here the centripetal acceleration is completely attributed to the kinetic energy term due to the absence of the relative vorticity term. Corresponding to Eq. 9b, the *x*-direction Rossby number is:

(10) $$Ro_{x}=\frac{v}{fr}$$

Evidently, the vorticity Rossby number *Ro_vort_* $=\;\frac{\zeta }{f}\;=\;0$ completely misrepresents the pointwise nonlinearity of the momentum equations in this case because of the neglect of the kinetic energy term.

Lastly, let us consider the special scenario where the kinetic energy is spatially uniform. Take, for instance, an idealized circular flow model with uniform kinetic energy (Fig. 1d). The momentum equations (i.e., Eq. 3) become:

(11) $$\left\{ \begin{array}{c} -\zeta v-fv=-g\frac{\partial \eta }{\partial x}\\ \zeta u+fu=-g\frac{\partial \eta }{\partial y} \end{array}\right.$$

The nonlinearity of Eq. 11 is completely determined by the relative vorticity term. Consequently, the pointwise Rossby numbers are totally explained by the relative vorticity as follows:

(12) $$Ro_{x}=Ro_{y}=\frac{\zeta }{f}=Ro_{vort}$$

Therefore, for the uniform-kinetic-energy circular flow, the vorticity Rossby number is perfectly exact in weighing the pointwise strength of the nonlinear advection term relative to the Coriolis acceleration. Unfortunately, this kind of flow seldom exists in the real ocean.

### 2.3 Short remark

It has been demonstrated in Sections 2.1-2.2 that the vorticity Rossby number fails to include the contribution of the kinetic energy term, which acts to counterbalance the relative vorticity term and is thus an incomplete measure for the relative strength of the advection and Coriolis terms. It is necessary to find an improved approximation of the pointwise Rossby number. At first sight, the simplest way of improvement appears to be retaining the kinetic energy term, namely directly utilizing *Ro_x_* and *Ro_y_* in Eq. 4 as a measure of the pointwise Rossby number. However, *Ro_x_* (*Ro_y_*) could become singular and incapable of providing an accurate description wherever the meridional (zonal) velocity is zero, and thus the Coriolis force vanishes. Instead of the conventional Cartesian coordinate, the natural coordinate system can be used to avoid such a singular case, which leads to the curvature Rossby number in Section 3.

## 3 Curvature Rossby number

### 3.1 Mathematical derivation

Consider the rotating shallow-water momentum equations in the natural coordinate system [4]:

$$\left\{ \begin{array}{cc} \frac{\partial V}{\partial t}+V\frac{\partial V}{\partial s}=-g\frac{\partial \eta }{\partial s} & \;\;\;\;\;\;\;\;\;\left(13a\right)\\ V\frac{\partial \alpha }{\partial t}+\zeta_{curv}V+fV=-g\frac{\partial \eta }{\partial n} & \;\;\;\;\;\;\;\;\;\left(13b\right) \end{array}\right.$$

where *s* (***s***) is the distance (unit vector) along the streamline, *n* (***n***) the distance (unit vector) perpendicular to the streamline, *α* the angular direction of the flow velocity and $\zeta_{\mathrm{curv}}=Vk\cdot \left(\nabla_{h}\times s\right)=\frac{V}{R_{s}}$ the curvature vorticity with *R_s_* denoting the radius of the streamline curvature. Recall that the sum of the curvature vorticity $\zeta_{\mathrm{curv}}$ and shear vorticity $\zeta_{\mathrm{shear}}=k\cdot \left(\nabla_{h}V\times s\right)=-\frac{\partial V}{\partial n}$ is equal to the relative vorticity ζ [13,14]. It is important to note that the Coriolis force is only present in the direction perpendicular to the streamline and that the nonlinear term along that direction is exactly the curvature vorticity term in contrast to the Cartesian formulation (i.e., Eq. 4) where the relative vorticity term only partially represents the nonlinearity. Therefore, it is straightforward to formulate the pointwise Rossby number in the direction perpendicular to the streamline by calculating the ratio of the second to third terms on the left-hand side of Eq. 13b, obtaining:

(14) $$Ro_{curv}=\frac{\zeta_{curv}}{f}=\frac{V}{fR_{s}}$$

As mentioned in Section 1, this formulation has been previously proposed and is here called the curvature Rossby number. Mathematically, *Ro_curv_* avoids the singularity induced by vanishing meridional or zonal velocity.

**Fig. 2:**
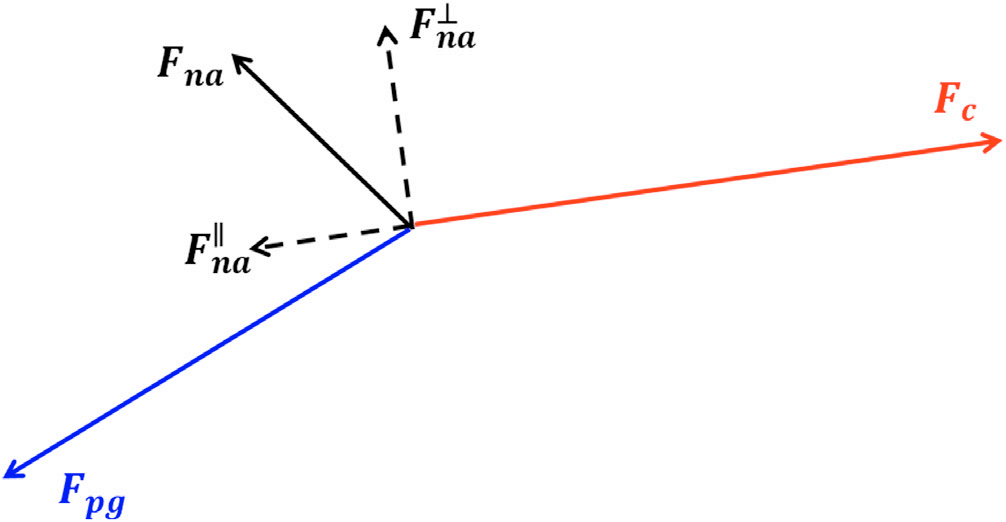
**A balance among the Coriolis acceleration**$F_{c}$**, pressure gradient**$F_{pg}$**and nonlinear acceleration**$F_{na}$**.**$F_{na}^{∥}$**(**$F_{na}^{⊥}$**) is the component of**$F_{na}$**parallel (perpendicular) to**$F_{c}$**.**

It is argued that *Ro_curv_* is an intrinsic description of the flow nonlinearity. For a physical explanation, let us invoke an argument that is independent of the coordinate system. Fig. 2 shows a balance among the Coriolis acceleration (i.e., $F_{c}=f\times u$ Where f=(0,0,f)) perpendicular to the streamline, pressure gradient (i.e., $F_{\mathrm{pg}}=g\nabla_{h}\eta$) and nonlinear acceleration (i.e., $F_{\mathrm{na}}=u\cdot \nabla_{h}u$). Two components of $F_{\mathrm{na}}$ are $F_{\mathrm{na}}^{∥}$ and $F_{\mathrm{na}}^{⊥}$, which are respectively parallel and perpendicular to $F_{c}$. When $F_{c}$ is used as a reference to weigh the importance of $F_{\mathrm{na}}$, it is precisely $F_{\mathrm{na}}^{∥}$ that is compared with $F_{c}$. It is readily proven that $F_{\mathrm{na}}^{∥}$ is exactly equal to:

(15) $$\begin{array}{ccc} F_{\mathrm{na}}\cdot F_{c}/\left|F_{c}\right| & = & \left(u\cdot \nabla_{h}u\right)\cdot \left(f\times u\right)/\left|f\times u\right|\\ & = & \left[\nabla_{h}\left(u\cdot u\right)/2+\zeta \times u\right]\cdot \left(f\times u\right)/\left|f\times u\right|\\ & = & \left[V^{2}s\times \nabla_{h}V+Vs\times \;\left(\zeta \times Vs\right)\right]\cdot f/\left(fV\right)\\ & = & \left[V^{2}s\times \nabla_{h}V+\left(Vs\cdot Vs\right)\zeta -\left(Vs\cdot \zeta \right)Vs\right]\cdot f/\left(fV\right)\\ & = & \left[V^{2}s\times \nabla_{h}V+\left(Vs\cdot Vs\right)\zeta \right]\cdot f/\left(fV\right)\\ & = & \left[V^{2}f\cdot \left(s\times \nabla_{h}V\right)+V^{2}\zeta \cdot f\right]/\left(fV\right)\\ & = & \left[k\cdot \left(s\times \nabla_{h}V\right)+\zeta \cdot k\right]V\\ & = & \left(-\zeta_{shear}+\zeta \right)V=\zeta_{curv}V \end{array}$$

*Ro_curv_* is exactly the ratio $F_{\mathrm{na}}^{∥}/F_{c}$ which is independent of the choice of the coordinate system and thus intrinsically measures the relative significance of the nonlinear acceleration and Coriolis force. It is also obvious from the derivation in Eq. 15 that the shear vorticity component of the relative vorticity term is completely cancelled by the kinetic energy term; consequently, the shear vorticity does not contribute to the nonlinearity of the momentum equation in the direction parallel to the Coriolis acceleration. Therefore, *Ro_curv_* correctly removes the spurious influence of the shear vorticity through recovering the kinetic energy term, while *Ro_vort_* redundantly includes the contribution of the shear vorticity due to the neglect of the kinetic energy term. Incidentally, it is unreasonable to weigh $F_{\mathrm{na}}^{⊥}$ against $F_{c}$ since they are completely misaligned. To determine the relative strength of $F_{\mathrm{na}}^{⊥}$, another reference term, such as the pressure gradient force along the streamline, can be used. But that is irrelevant to the formulation of the pointwise Rossby number and thus beyond the scope of this study.

To summarize, *Ro_curv_* is superior in describing the pointwise nonlinearity strength relative to the Coriolis force, which will be further illustrated by idealized flow models in the following.

### 3.2 Idealized flow models

Table 1 shows the vorticity Rossby number *Ro_vort_* and the curvature Rossby number *Ro_curv_* for the four idealized flow models whose exact pointwise Rossby numbers (i.e., *Ro_x_* or/and *Ro_y_*) at the red-pentagram points (Fig. 1) are known *a priori*. As also detailed in Section 2, *Ro_vort_* completely misrepresents the pointwise Rossby number for the typical oceanic shear flow with zero curvature vorticity but non-zero shear vorticity and it overestimates the nonlinearity of the typical oceanic vortex whose shear vorticity and curvature vorticity are of the same sign, which holds regardless of the vortex polarity and hemisphere. Only for the uniform-kinetic-energy circular flow whose shear vorticity vanishes, *Ro_vort_* correctly reproduces the pointwise Rossby number. By contrast, *Ro_curv_* faithfully recovers *Ro_x_* or/and *Ro_y_* for all idealized flow models.

**Table 1:** **Rossby numbers for the four idealized flow models**.

	Uniform shear flow	Uniform circular flow	Point vortex	Uniform kinetic energy flow
*Ro_x_*	*Ro_x_ is ill-defined*	$Ro_{x}=\frac{v}{fr}$	$Ro_{x}=\frac{v}{fr}$	$Ro_{x}=\frac{\zeta }{f}=\frac{\zeta_{curv}}{f}$
*Ro_y_*	$Ro_{y}=0$	*Ro_y_ is ill-defined*	*Ro_y_ is ill-defined*	$Ro_{y}=\frac{\zeta }{f}=\frac{\zeta_{curv}}{f}$
*Ro_vort_*	$\frac{A}{f}$	$\frac{2v}{fr}$	0	$\frac{\zeta }{f}=\frac{\zeta_{curv}}{f}$
*Ro_curv_*	0	$\frac{v}{fr}$	$\frac{v}{fr}$	$\frac{\zeta_{curv}}{f}$

Note that $\zeta_{shear}=0$ for the uniform kinetic energy flow.

## 4 Quasi-realistic application

**Fig. 3:**
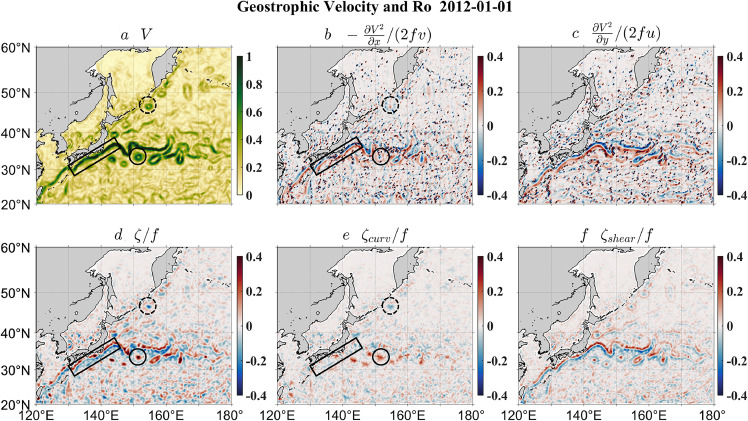
**The satellite-altimeter-based flow speed (a), the ratio of the*****x*****-derivative of the kinetic energy to the*****x*****-direction Coriolis acceleration (b), the ratio of the*****y*****-derivative of the kinetic energy to the*****y*****-direction Coriolis acceleration (c), the ratio of the relative vorticity to the planetary vorticity (d), the ratio of the curvature vorticity to the planetary vorticity (e) and the ratio of the shear vorticity to the planetary vorticity (f) around the Kuroshio and its extension.** The black boxes, black solid circles and black dashed circles highlight a shear flow, a cyclonic vortex and an anticyclonic vortex, respectively.

**Fig. 4:**
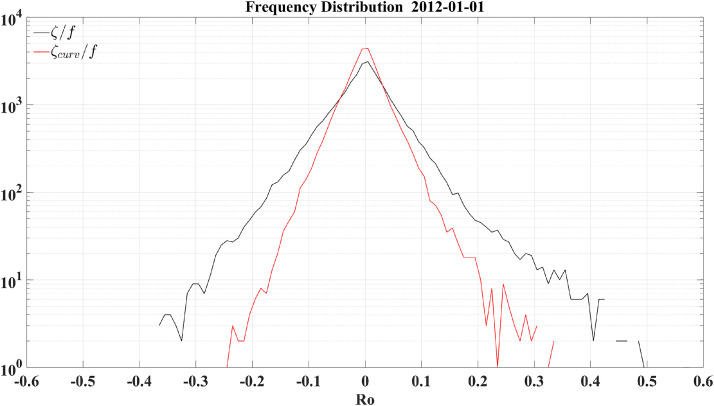
**The satellite-altimeter-based frequency distribution of the Rossby numbers.** The black and red curves denote the vorticity and curvature Rossby numbers, respectively. Note that a logarithmic scale is used for the *y* axis.

With the mathematical derivation and idealized flow models having provided solid evidence in 2, 2.1, we now proceed with the quasi-realistic cases. First, the gridded geostrophic velocity data are used to compare the vorticity and curvature Rossby numbers. The data are derived from sea surface height observations by the nadir-looking satellites and provided by the Copernicus Marine Service. The spatial resolution of the data is 1/4^°^, and thus large-scale motions are well captured, and mesoscale processes are partially resolved. Fig. 3 shows the comparison around the Kuroshio and its extension, a region characterized by shear flows (e.g., the black box in Fig. 3a) and mesoscale vortices (e.g., the solid and dashed circles in Fig. 3a, respectively). The vorticity Rossby number *Ro_vort_* (Fig. 3d) has large magnitudes along the shear flow and inside vortices, while the kinetic energy terms (Fig. 3b, c) tend to have a counteracting effect, especially along the shear flow. Note that the distribution of the kinetic energy term has a patchy structure of extremely large/small values resulting from the nearly vanishing meridional or zonal velocity. These singular points indicate that, as argued in Section 2, a direct inclusion of the kinetic energy term into the vorticity Rossby number is not the proper way to improve the representation of the pointwise Rossby number. On the other hand, the curvature Rossby number *Ro_curv_* (Fig. 3e), correctly excluding the contribution of the shear vorticity (Fig. 3f), especially along the shear flow and inside vortices, is generally smaller in magnitude. This is more clearly shown by the histograms of *Ro_vort_* and *Ro_curv_* in Fig. 4, with the former displaying a wider distribution than the latter. Such an overestimate of the nonlinearity by *Ro_vort_* is understandable since the shear vorticity tends to be of the same sign as the curvature vorticity, as obviously seen in the case of the oceanic mesoscale vortex. The vanishingly small *Ro_curv_* along the shear flow and decreased *Ro_curv_* inside mesoscale vortices agree with the basic observations from idealized flow models in Section 3.2. In addition to the magnitude overestimate, the standard deviation of *Ro_curv_* (i.e., 0.042) is 42% smaller than that of *Ro_vort_* (i.e., 0.072), indicating that the distribution patterns of the two Rossby numbers are also strikingly different.

**Fig. 5:**
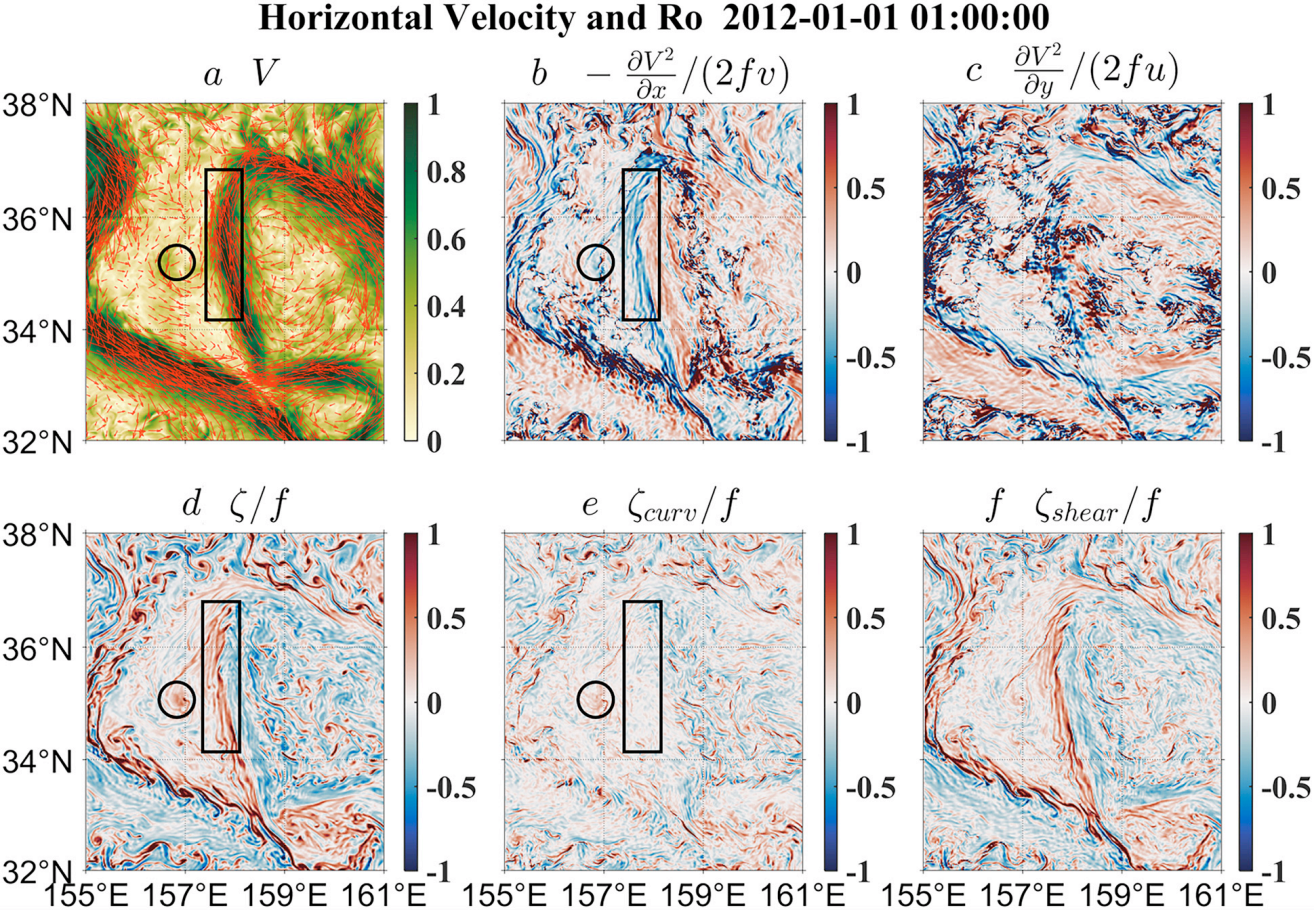
**The LLC4320 flow velocity and speed (a), the ratio of the*****x*****-derivative of the kinetic energy to the*****x*****-direction Coriolis acceleration (b), the ratio of the*****y*****-derivative of the kinetic energy to the*****y*****-direction Coriolis acceleration (c), the ratio of the relative vorticity to the planetary vorticity (d), the ratio of the curvature vorticity to the planetary vorticity (e) and the ratio of the shear vorticity to the planetary vorticity (f) around the Kuroshio Extension.** The black boxes and circles highlight a shear flow and a cyclonic vortex, respectively.

**Fig. 6:**
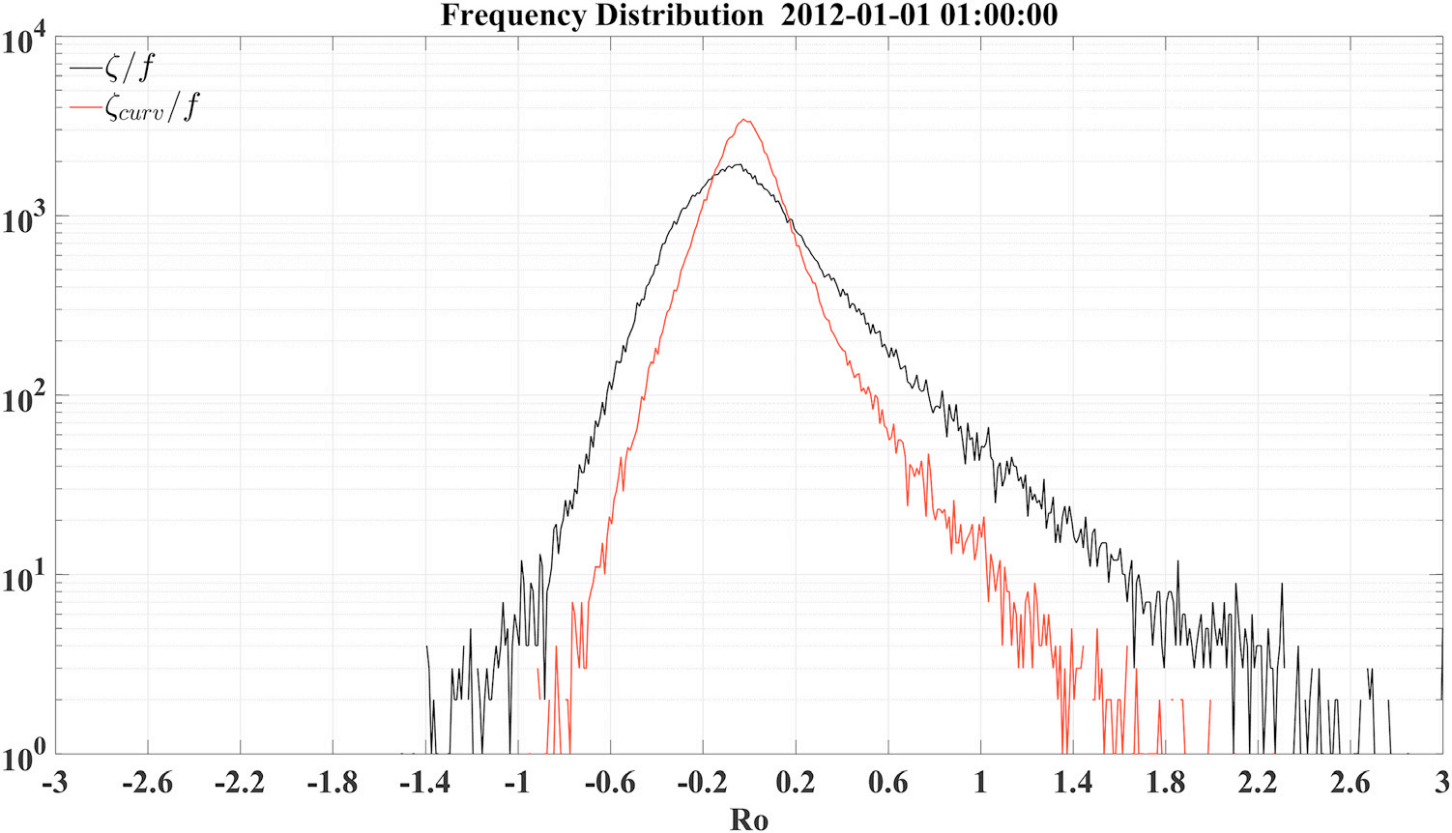
**The LLC4320-based frequency distribution of the Rossby numbers.** The black and red curves denote the vorticity and curvature Rossby numbers, respectively. Note that a logarithmic scale is used for the *y* axis.

Since nadir-looking satellite altimetry cannot well resolve submesoscale flows and the internal gravity wave continuum, a realistic numerical simulation (i.e., LLC4320) is employed to further demonstrate the advantages of the curvature Rossby number. Simultaneously forced by atmospheric fields and tidal potentials, the LLC4320 simulation has a spatial resolution of ∼2 km and outputs hourly snapshot variables. This simulation can reproduce not only large-scale currents and mesoscale variabilities [15,16] but also submesoscale flows and internal gravity waves [[17], [18], [19]], mimicking the real ocean with multiscale dynamical processes. We focus on a section of the Kuroshio Extension (Fig. 5), especially on a shear flow (black boxes) and a cyclonic vortex (black circles). According to the vorticity Rossby number *Ro_vort_* (Fig. 5d), the shear flow and the eddy display seemingly strong nonlinearity, which is, however, counteracted by the kinetic energy term (Fig. 5b-c). This is consistent with the conclusion from the idealized flow model in Section 2.2 and the satellite observation in Fig. 3. Again, the kinetic energy term should not be directly added to estimate the pointwise Rossby number because of its unreasonably high/low values resulting from velocity singularity. By contrast, the curvature Rossby number *Ro_curv_* (Fig. 5e) excludes the spurious contribution of the shear vorticity (Fig. 5f) contained in the vorticity Rossby number *Ro_vort_*, and thus correctly exhibits a much smaller magnitude than *Ro_vort_*, which is also evident in the histograms of *Ro_vort_* and *Ro_curv_* (Fig. 6). Thus *Ro_curv_* presents a nearly negligible nonlinearity for the shear flow and a much weaker nonlinearity for the cyclonic vortex shown in Fig. 5, which agrees with the results from the uniform shear flow and the uniform circular flow in Section 3.2. In addition to the obvious difference in the magnitude, *Ro_curv_* and *Ro_vort_* also show quite distinct distribution patterns, as demonstrated by their contrasting standard deviations (0.202 for *Ro_curv_* and 0.348 for *Ro_vort_*).

## 5 Summary and discussion

This short study revisits two approximate formulations of pointwise Rossby numbers, largely motivated by the recent attention on submesoscale processes where the nonlinearity is strong and comparable to the effect of the Earth's rotation. Most often, the pointwise Rossby number is approximated by the vorticity Rossby number, which is the ratio of the relative vorticity to the planetary vorticity. The vorticity Rossby number fails to include the effect of the kinetic energy term, as seen when the momentum equations are presented in the Gromeka-Lamb form. Using idealized flow models, it is demonstrated that the kinetic energy term is an important contributor to the nonlinearity and definitely non-negligible. As an alternative, the curvature Rossby number was previously proposed as the ratio of the curvature vorticity to the planetary vorticity and, by construction, serves as a better candidate for the pointwise Rossby number owing to the recovery of the kinetic energy term. The application to idealized flow models and quasi-realistic oceanic data further confirms that the curvature Rossby number is a more faithful representation of the relative magnitude of the nonlinear advection and the Coriolis force, while the vorticity Rossby number redundantly includes the contribution of the shear vorticity and thus significantly overestimates the pointwise nonlinearity of the momentum equations. In particular, the curvature Rossby number correctly estimates the pointwise Rossby number of the uniform shear flow to be zero while the vorticity Rossby number gives a complete misrepresentation; the curvature Rossby number accurately estimates the pointwise Rossby number of the idealized oceanic vortex while the vorticity Rossby number formulation gives an overestimate by a factor of 2. It is expected that the curvature Rossby number, which represents a perspective from the natural coordinate system, will help us to diagnose and understand the multiscale ocean dynamics in general and the highly-nonlinear submesoscale processes in particular. Coincidentally, ocean dynamics in the natural coordinate system has recently attracted much attention and revealed illuminating insights into the cyclogeostrophic adjustment and frontogenesis of a curved front [20], the Ekman transport and pumping in a curved balanced flow [21], the symmetric instability of a curved front [22,23], etc. It seems that the curved ocean dynamics merits more future explorations.

## Data availability statement

The satellite altimeter data can be downloaded from https://data.marine.copernicus.eu/product/SEALEVEL_GLO_PHY_L4_MY_008_047/description. The LLC4320 simulation outputs are available at https://data.nas.nasa.gov/ecco/data.php?dir=/eccodata/llc_4320. The code to calculate the curvature Rossby number is available at https://doi.org/10.5281/zenodo.10578948.

## Declaration of competing interest

The authors declare that they have no conflicts of interest in this work.
